# The Rurikids: The First Experience of Reconstructing the Genetic Portrait of the Ruling Family of Medieval Rus’ Based on Paleogenomic Data

**DOI:** 10.32607/actanaturae.23425

**Published:** 2023

**Authors:** K. V. Zhur, F. S. Sharko, Vl. V. Sedov, M. V. Dobrovolskaya, V. G. Volkov, N. G. Maksimov, A. N. Seslavine, N. A. Makarov, E. B. Prokhortchouk

**Affiliations:** Federal Research Centre “Fundamentals of Biotechnology” of the Russian Academy of Sciences, Moscow, 119071 Russian Federation; Institute of Archeology, Russian Academy of Sciences, Moscow, 117292 Russian Federation; Regional State Autonomous Institution “Center of Tatar Culture”, Tomsk, 634050 Russian Federation; ANO “Runiverse”, Moscow, 119071 Russian Federation; Russian Public Organisation “RDS”, Moscow, 109028 Russian Federation

**Keywords:** the Rurikids, Prince Dmitry Alexandrovich, whole genome sequencing, N1a-haplogroup

## Abstract

The Rurikids were the reigning house of Rus’, its principalities and, ultimately the Tsardom of Russia, for seven centuries: from the IX to the end of the XVI century. According to the Primary Chronicle (the Tale of Bygone Years), the main chronicle of Rus’, the Rurik dynasty was founded by the Varangian prince Rurik, invited to reign in Novgorod in 862, but still there is no direct genetic evidence of the origin of the early Rurikids. This research, for the first time, provides a genome-wide paleogenetic analysis of bone remains belonging to one of the Rurikids, Prince Dmitry Alexandrovich (?–1294), the son of the Grand Prince of Vladimir Alexander Yaroslavich Nevsky (1221–1263). It has been established that his Y chromosome belongs to the N1a haplogroup. Most of the modern Rurikids, according to their genealogies, belonging to the N1a haplogroup, have the most similar variants of Y chromosomes to each other, as well as to the Y chromosome of Prince Dmitry Alexandrovich. Genome-wide data of the medieval and modern Rurikids unequivocally indicates that they belong to the N1a haplogroup of the Y chromosome, starting at least from the XI century (since the time of Prince Yaroslav the Wise). All the other alleged Rurikids, both ancient and modern, being carriers of other haplogroups (R1a, I2a), possess high heterogeneity of the sequence of Y chromosomes, meaning that we cannot confirm their common ancestry. The most probable ancestors of Prince Dmitry Alexandrovich in the male line were the men who left the burial ground Bolshoy Oleny Island on the coast of the Kola Peninsula about 3,600 years ago. The reconstruction of the genome of Prince Dmitry Alexandrovich indicates the contribution of three ancestral components to his origin: (1) the early medieval population of the east of Scandinavia from the island of Oland, (2) representatives of the steppe nomadic peoples of the Eurasian steppes of the Iron Age or the early medieval population of central Europe (steppe nomads from the territory of Hungary), and (3) the ancient East-Eurasian component. Reliable statistics were also obtained when the Scandinavians were replaced with the Medieval Russian Slavic populations of the XI century. Thus, for the first time, we have shown the complex nature of interethnic interactions in the formation of the nobility of medieval Rus’ on the example of the ancient Rurikid.

## INTRODUCTION

The application of paleogenetic methods when studying the genetic identity and origin of the medieval Russian nobility is one of the most productive among many modern scientific approaches capable of expanding and verifying the existing knowledge about the Medieval Russian society, its ethnic composition, and political organization. Meanwhile, the remains of the Rurikids, the most ancient reigning family whose members were the major actors in the history of Russia in the IX–XVI centuries, remain almost untouched by paleogenetic researches. The XI– XII centuries Rurikids’ haplogroups were reconstructed based on genetic materials of modern individuals whose genealogy, according to historical data, ascends to Rurik with different degrees of reliability [1]. The accuracy in the selection of these genetic materials and the possibility of verification of the historical and genealogical information on the basis of which the selection was made remain debatable and are discussed by the authors – experts of absolutely different qualifications and fields [2, 3].

The existence of a “blind spot” in the study of the genomes of the Medieval Russian nobility in many aspects is due to the complexity of personally identifying the remains of the Rurikids and other aristocratic families in the necropolises of the X–XIV centuries. It is well known that the names of the buried were not indicated in any way on funerary structures, sarcophagi, or tombstones until the beginning of the XV century. The location of princely burial grounds is established by annalistic messages, synodics of the XVI–XVII centuries, taking into account the later tradition of church veneration of many representatives of the princely family. Archaeological research of burial grounds in Medieval Russian churches and the anthropological study of bones are the main ways of identifying noble burials, but the conditions of necropolises do not always allow for such identification. The long use of necropolises, the practice of placing new burials over old ones, moving the revered remains during their examination in the XV–XIX centuries, and, finally, the removal of relics in the course of the anti-religion campaigns in Soviet times led to part of the princely remains of the XI–XIV centuries from the burial places in the Medieval Russian churches getting lost, or the remains not being reliably matched with certain historical persons whose graves were disposed in these necropolises. One example of the use of bone remains in order to conduct the genetic analysis of the Rurikids whose membership in the princely ruling family cannot be verified by archaeological data is the study of the presumed remains of Prince Gleb Svyatoslavich from the Chernigov Transfiguration Cathedral – a skull found during repair works at the temple without archaeological documentation [4].

Thus, the few burial places of the Rurikids with bone remains that can be reliably attributed to the princely family, based on the archaeological data, anthropological definitions, and a set of historical evidence acquire special significance. To such trustworthy burial places belongs the burial site of prince Dmitry Alexandrovich, found in the southern altar apse in the south-eastern part of the Transfiguration cathedral in Pereslavl-Zalessky (Supplementary 1).

## EXPERIMENTAL PART

**DNA isolation and genomic library preparation**

All experiments with aDNA were carried out in “a clean room” – a room specially equipped for these purposes at the Federal Research Center “Fundamentals of Biotechnology” of the Russian Academy of Sciences (Skryabin Institute of Bioengineering).

DNA was isolated from the bone remains found in the ruined sarcophagus of the Transfiguration cathedral in Pereslavl-Zalessky. According to the historical information on the burial place, archeological data and anthropological definitions, these remains belong to the son of Prince Alexander Yaroslavich Nevsky – Prince Dmitry Alexandrovich (Supplementary 1). The remains are characterized by good preservation of bone tissue, which is typical for remains found long after burial out of contact with the ground, suggesting a rather late episode of destruction of the sarcophagus. To isolate aDNA from the samples provided for genetic analysis, we obtained three portions of bone powder weighing 20, 50, and 80 mg from the metacarpal bone of the hand, rotula, and navicular bone of the foot, respectively. DNA was isolated by magnetic separation using buffer D – that of the Dabney method (5 M guanidine hydrochloride, 40% (v/v), isopropanol, 0.12 M sodium acetate, and 0.05% (v/v) Tween 20) and silica-coated magnetic beads [5].

The resulting DNA was used to prepare libraries of single-stranded DNA fragments using the ACCEL-NGS 1S Plus DNA Library Kit (Swift Biosciences, USA) according to the original protocol but with minor modifications: for the steps providing strand elongation and sample indexing, uracil-tolerant polymerase (KAPA HiFi HS Uracil+RM, USA) was used. To assess the content of endogenous DNA, test sequencing of the constructed libraries of lowcoverage DNA fragments was carried out, approximately 3–4 million single reads per sample (50 bp long). For the sample with the best preservation of the genetic material (high endogeneity and the presence of C > T substitutions at the 5’ ends of DNA fragments), an additional library was prepared from the same DNA extract and pre-treated with a mixture of uracil-DNA glycosylase (UDG) and endonuclease VIII [6]. The mixture of enzymes made it possible to remove uracil from the aDNA strands and turn the resulting abasic sites into single nucleotide breaks, while some of the uracils at the ends of the fragments were preserved, which is associated with the low efficiency of enzymes in these regions. The removal of uracils improved the quality of mapping and prevented a distortion of the results of the subsequent statistical processing [7].

The MyBaits Expert Human Affinities Prime Plus Kit (Daicel Arbor Biosciences) was used for subsequent enrichment of the genome regions of interest. Biotinylated single-stranded DNA probes from the kit cover single nucleotide polymorphisms (SNPs) from the panel “1240K capture” [8], 46,000 additional unique SNPs of the Y chromosome of known haplogroups according to the classifier of the International Society of Genetic Genealogy (ISOGG ) [9], and a set of MitoTrio probes for three different mitochondrial genomes: the Revised Cambridge Reference Sequence (rCRS), the Reconstructed Sapiens Reference Sequence (RSRS), and the Vindija Neanderthal sequence (Genbank NC_011137) [10]. Libraries were sequenced on a HiSeq 1500 instrument (Illumina, USA) in paired read mode 2 × 150 bp for genome-wide sequencing and in the mode of single readings 50 bp long for test libraries.

**Bioinformatics analysis**

To remove contaminating DNA reads from the sequencing data, we used the BBDuk software [11] included in the BBMap package, and bacteria, fungi, plants, viruses, and other organism databases. The output of the BBDuk tool was analyzed using the PALEOMIX pipe-line (version 1.2.14) [12]. Sequencing adapters were trimmed using the Cutadapt v3.4 tool [13]. Sequences were aligned to the reference human genome sequence (hg19/GRCh37) using BWA (version 0.7.17) [14].

Aligned reads were filtered to ensure a minimum display quality of 20 using samtools view (version 1.9) [15]. Indexing, sorting, and removal of duplicates (rmdup) were performed using the samtools tool (version 1.9) [15]. To call genotypes from aligned reads, a PileupCaller (https://github.com/stschiff/sequenceTools) with the “–randomHaploid” mode was used, which calls haploid genotypes by randomly selecting one high-quality base (phred base quality score ≥ 30) on the 1240K SNP panel (https://reich. hms.harvard.edu/).

Postmortem DNA damage patterns were analyzed using the MapDamage2 software [16], which offers a series of tools for imaging and modeling postmortem damage patterns observed in ancient samples. MapDamage2.0 also makes it possible to recalculate base quality scores in order to mitigate the impact of postmortem damage on further analysis.

To determine the genetic clustering of the NEV2.3 sample among the ancient samples known at the time of the study presented in the Allen Ancient DNA Resource (AADR) panel [17], the ADMIXTURE v.1.3.0 software [18] was used. SNPs were trimmed for sites with linkage disequilibrium using PLINK v1.9 [19]. The sliding window was 50 SNPs; the step was 5 SNPs; the r2 threshold was 0.2 (–in-dep-pairwise 50 5 0.2). There were 10 runs with random starting values for a number of clusters (*K*) in the range of 4–12; the run with the lowest cross-validation error was selected to plot the graph of population admixture.

For principal component analysis (PCA), the smartpca tool from the EIGENSOFT package was used. Ancient samples were projected onto the first two components of the modern samples. A list of samples is presented in *Table 1* of Supplementary 2. The following parameters were set by default: lsqproject: YES, numoutlieriter; 0, shrinkmode; and YES for the smartpca analysis. Mitochondrial haplotypes were determined using the HaploGrep program [20]. Determination of Y chromosome haplogroups was carried out by comparing alleles on the phylogenetic tree ISOGG version 15.73. F4-statistics were calculated using the qpDstat program from the ADMIXTOOLS software package with default parameters. All constructions were based on available data obtained from whole genome sequencing of the samples. To model the genome from the components of ancestral populations, we used the qpWave and qpAdmix programs with the “allsnps: YES” parameter and “Russia_Yana_UP,” “Russia_Sunghir,” “Bichon_ LP,” “Zagros_EN,” “Russia_DevilsCave_N,” “Alaska_ LP,” “Russia_Ust_Ishim.DG,” “Papuan.DG,” “Han.DG,” “Chukchi.DG,” “Russia_Kostenki14,” “ONG,” “Yoruba. SDG,” “Mbuti.SDG,” and “Karitiana.SDG” were chosen as the right populations.

The HIrisPlex-S online tool [21, 22, 23] was used to predict eye, hair, and skin color.

**DNA isolation and genomic library preparation from a modern human blood sample**

To avoid the bias caused by the method of library preparation, we sequenced a sample of a modern Rurikid (sample with an identifier Olgovich3), according to his genealogy. Informed consent to participate in the genetic research was obtained from the modern Rurikid before the start of any research procedures.

Genomic DNA was isolated from 200 μl of blood using a Magen DNA blood mini kit according to the protocol. Overall, 1 μg of genomic DNA was used to fragment the sample to an average DNA fragment size of 200 nucleotides on a Covaris platform. The library for subsequent whole genome sequencing was prepared according to the instructions in the NEBNEXT DNA UltraII (NEB) kit. Sequencing was carried out on a HiSeq1500 platform (Illuimina, USA). In total, 115,564,028 reads of 150 nucleotides were generated. Mapping was performed using BWA (v0.7.17) on the hg19/GRCh37 reference human genome, followed by the removal of PCR duplicates. For further identification of SNPs, we used 104,109,318 reads generated on the bcftools software.

Genetic testing of the alleged modern Rurikids was carried out in the commercial laboratory FamilyTreeDNA in Houston (USA), in the laboratory of human population genetics at the Research Centre of Medical Genetics named after the Academician N.P. Bochkov (Moscow) and in the laboratory of evolutionary genetics at the Research institute of medical genetics (Tomsk). The results of the genetic testing were provided by Seslavin A.N., Volkov V.G. and Maksimov N.G., the leaders of the international research project “Rurikovichi. The genome of Russian princes.” Some of the results were presented as bam files (Olgovich1, Yurievich1, Mstislavich1, Mstislavich2, Yurievich2, Olgovich4, Mstislavich3, Mstislavich4, Yurievich3, Mstislavich5), part as a list of SNPs of the Y chromosome (Olgovich2 and Olgovich5). All participants donated their genetic data to be used in this project.

## RESULTS AND DISCUSSION

**Discovery of the remains of Prince Dmitry Alexandrovich in the Transfiguration Cathedral in Pereslavl-Zalessky**

The architectural and archaeological team of the Institute of Archeology of the Russian Academy of Sciences, led by V.V. Sedov, examined the alleged burial site of Prince Dmitry Alexandrovich in Pereslavl-Zalessky. Prince Dmitry Alexandrovich (?–1294) was the second son of Prince Alexander Yaroslavich, who inherited the Principality of Pereslavl after his father’s death (1263). At different times Prince Dmitry possessed the Novgorod and the great Vladimir principalities. He died on Volok Lamsky on his way to Pereslavl from Tver, and he was buried in Pereslavl (Complete collection of Russian chronicles, vol. 1, 282 p.; Complete collection of Russian chronicles, vol. 3, 328 p.). At the same time, a number of chronicles (including the 4^th^ Novgorod chronicle, the Moscow chronicle of the late XV century, Voskresenskaya and Nikonovskaya chronicles) contain direct references to the fact that he was buried in the Transfiguration Cathedral of Pereyaslavl-Zalessky (Complete collection of Russian chronicles, vol. IV, 249 p.; Complete collection of Russian chronicles, vol. XXV, 157 p.; Complete collection of Russian chronicles, vol. VII, 181 p.; Complete collection of Russian chronicles, vol. XIII, 170 p.) [24, 25, 26, 27, 28]. The identification of the remains from the sarcophagus in the southwestern part of the cathedral as the burial place of Prince Dmitry Alexandrovich is based on a combination of historical information on the burial, archaeological data, and anthropological definitions (Supplementary 1).

**Paleogenetic analysis of bone remains from the sarcophagus**

To isolate aDNA, bone samples (metacarpal, patella, and navicular bone of the foot) of an adult individual, presumably Prince Dmitry Alexandrovich (identification number Nev2), were collected. Bone powder weights were obtained from the samples with the corresponding identification numbers Nev2.1, Nev2.2, and Nev2.3, from which DNA was isolated and libraries of single-stranded fragments were prepared for the initial shotgun sequencing in order to assess endogeneity. Sequencing results are presented in Supplementary 3.

The Nev2.3 sample was characterized by the highest proportion of endogenous DNA and the frequency of cytosine to thymine substitutions at the 5’ ends of aDNA fragments and was selected for further in-solution enrichment and sequencing. The frequency of C to T substitutions around the 5´ ends of the sequences are presented in the figure in Supplementary 4.

As a result of genome-wide sequencing of the library of Nev2.3 aDNA fragments, more than 15 million reads were generated and 532,154 single nucleotide polymorphisms (SNPs) were identified. The data suggested that the genome belonged to a man, his mitochondrial haplogroup was F1b, and the Y chromosome haplogroup was N1a (*Table 1*). As a result of assessing the level of contamination of the sample according to such parameters as the degree of heterozygosity of mtDNA and X chromosome (*Table 1* and *Table 2* of Supplementary 5), no contamination of the sample was detected.

**Table 1:** Characteristics of the Nev2.3 sample sequencing

ID of the library	Original number of reads	Number of reads after filtering	Number of reads mapped on hg19	After removal of PCR duplicates	Coverage	Endogenous of DNA, %	SNP (for analysis)	Genetic gender	mtDNA haplogroup	Y chromosome haplogroup
NEV_2.3	15001647	14976811	14299210	3025176	0.06	20.2	532154	M	F1b1	N1a1a1a1a1a1a7a~

**Prediction of the phenotypic traits of Prince Dmitri Alexandrovich based on genetic data**

**Table 2:** Results of the prediction of the phenotypic traits of sample NEV2.3

Trait	Probability (P)
Brown eye color	0.962
Dark hair color	0.810
Intermediate skin tone	0.635
Brown hair color	0.555
Black hair color	0.355
Fair skin	0.306
Light hair color	0.190
Light blond hair color	0.090
Dark skin	0.053
Intermediate eye color	0.035
Very light skin	0.005
Blue eye color	0.003
Very dark or black skin tone	0.001
Red hair color	0.000

We have investigated the sample NEV2.3, presumably belonging to Prince Dmitry Alexandrovich, son of Prince Alexander Yaroslavich Nevsky, and managed to identify the single-nucleotide polymorphisms that allow us to predict his phenotype with a reasonable probability: hair color, skin color, and eye color. Eye color was most likely to be brown (*P *= 0.962), hair color was dark (*P *= 0.810) or brown (*P *= 0.555), and skin tone was intermediate (0.635), that is, neither light nor dark. Prediction results for the phenotypic properties of sample NEV2.3 are shown in *Table 2*.

**Analysis of the Y chromosome sequences of Prince Dmitry Alexandrovich and other alleged later representatives of the Rurik family**

There are three possible variants of the Y chromosome haplogroup of Rurik and his descendants – N1a, R1a, and I2a. The hypotheses are based on the results of genetic studies of the alleged 43 modern Rurikids (who are representatives of 32 genera from different branches of the alleged direct descendants of Prince Yaroslav the Wise and the Polotsk Rurikids) and three ancient descendants of Rurik [1, 2, 29, 30, 31, 32].

For the phylogenetic positioning of the Y chromosome, we used all the samples available in the Allen Ancient DNA Resource (AADR) database [17] carrying the haplogroup N1a, as well as the results of genotyping of the Y chromosomes of modern Rurikids with a similar haplogroup. The analysis did not aim at establishing a high-resolution haplogroup, but we concentrated on the analysis of all established polymorphisms of the Y chromosome of the sample (51017 SNP, Supplementary 6).

**Fig. 1:**
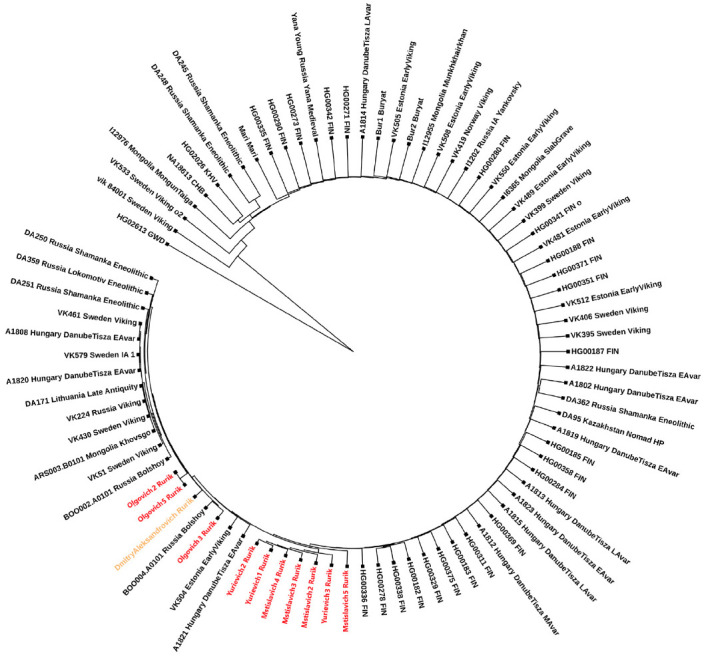
Phylogenetic tree of the Y chromosome, reflecting the family relationships between Prince Dmitry Alexandrovich (the sample is marked in orange), modern representatives of the Rurikids (marked in red), and samples from the database “The Allen Ancient DNA Resource,” belonging to the haplogroup N1a

As a result of phylogenetic positioning, the Y chromosome of Prince Dmitry Alexandrovich was clustered together with the Y chromosomes of alleged modern Rurikids (*Fig. 1*) originating from various noble families: Mstislavich2 (M), Mstislavich3 (M), Mstislavich4 (M), Mstislavich5 (M), Yurievich1 (Yu), Yurievich2(Yu), Yurievich3 (Yu), Olgovich2 (O), Olgovich3 (O), and Olgovich5 (O). M stands for the Mstislaviches, heirs of the branch of the princely Monomakh family descended from Prince Mstislav Vladimirovich (1076–1132); Yu, for the Yurieviches, a branch of the Rurikids derived from the great prince of Kiev Yurii Dolgorukii († 1157); and O, for the Olgoviches, the heirs of the middle line of Chernigov princes, the descendants of Oleg “Gorislavich” Svyatoslavich († 1115). Hereinafter we will use the symbols M, O, and Yu (these branches are reproduced in Supplementary 7) in order to attribute the samples to the genealogic branch of Rurikids. The detailed genealogy of the Rurikids is provided in Supplementary 8.

The three ancient alleged Rurikids, whose Y chromosome haplogroups were previously determined by other scientific groups, include a sample allegedly belonging to Prince Gleb Svyatoslavich of Chernigov (O), published under the identification number VK542 [4], a sample presumably belonging to Prince Izyaslav Ingvarevich Lutsky (M) with the identification number VK541 [4], and a sample belonging to Bela Rostislavovich (O), a large Hungarian feudal lord, a representative of the Chernigov line of the princely family of the Rurikids [33]. The Y chromosomal haplogroups established for these samples are as follows: Prince Gleb – I2a (whole genome sequence); Prince Izyaslav – R1a (whole genome sequence); and Prince Bela – N1a1a1a1a1a1a (according to STR markers). It is important to note that the belonging of the Chernigov and Lutsk burial places to the Rurikids cannot be substantiated by archaeological data, which calls into question the hypotheses that follow from the genetic analysis of these samples.

**Fig. 2:**
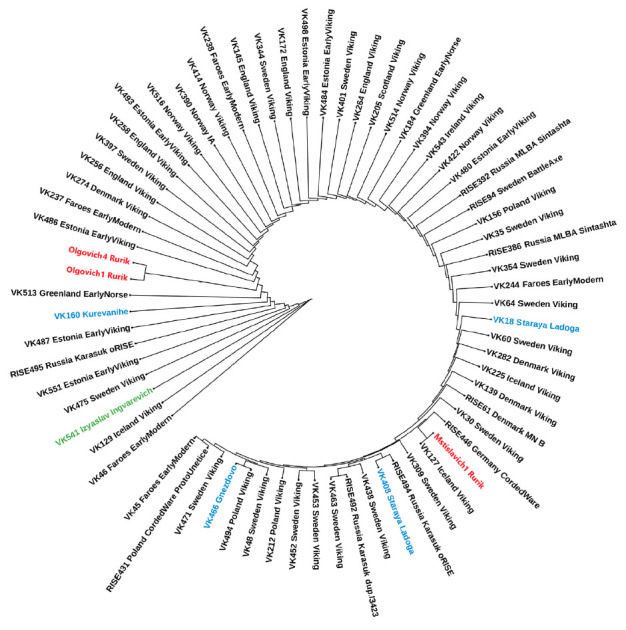
Phylogenetic tree of the Y chromosome, reflecting the relationship between the alleged modern Rurikids (marked in red) and samples from the database “The Allen Ancient DNA Resource,” belonging to the haplogroup R1a. The green color marks the sample of the alleged Prince Izyaslav Ingvarevich Lutsky, the blue color marks the samples of “Vikings” from the territory of modern Russia

For carriers of the haplogroup R1a of the modern representatives of the Rurikids and samples from the AADR database, an analysis was carried out using the same algorithm as for carriers of the haplogroup N1a (*Fig. 2*). It turned out that the Y chromosome of the alleged prince Izyaslav Ingvarevich Lutsky, although it belongs to the haplogroup R1a, does not cluster together with the samples of contemporary representatives of the Rurikids: the samples of Mstislavich1 (M), Olgovich1 (O), and Olgovich4 (O). Moreover, Mstislavich1 is clustered separately from Olgovich1 and Olgovich4. We should mention that these samples do not cluster with other “Vikings” with haplogroup R1a whose remains were found in Gnezdovo (VK466), Staraya Ladoga (VK408, VK18), and Kurevanikha (VK160) [4].

Thus, all modern descendants of the legendary Prince Rurik (according to their pedigrees) belonging to the N1a haplogroup and Prince Dmitry Alexandrovich have highly similar Y chromosomes. The aggregate of genome-wide data on the medieval and modern Rurikids unequivocally indicates that they belong to the N1a haplogroup of the Y chromosome, starting at least from the XIth century (since the time of Prince Yaroslav the Wise). All the other prospective Rurikids, both ancient and modern, being carriers of other haplogroups (R1a, I2a), possess high heterogeneity of the sequence of Y chromosomes; we cannot, therefore, confirm their common ancestry.

**Search for archaeological samples with the Y chromosome sequences closest to Prince Dmitry Alexandrovich**

The Y chromosome of Prince Dmitry Alexandrovich, in addition to the modern Rurikids, is clustered in the same branch with the ancient people from Bolshoy Oleny Island (Russia_Bolshoy), a burial ground dating back to the middle of the 2^md^ millennium BC, located in the Kola district of the Murmansk region (*Fig. 1*). Previously, using these samples as an example, the gene flow of the peoples of Siberia (East Eurasian component) to the North and East of Europe was shown [34]. A high degree of homology in the Y chromosome of a representative of the Russian noble family and people of the early metal era led us to the hypothesis of the possible contribution of the East Eurasian gene pool to the formation of the northern European population of the early Middle Ages.

**Fig. 3:**
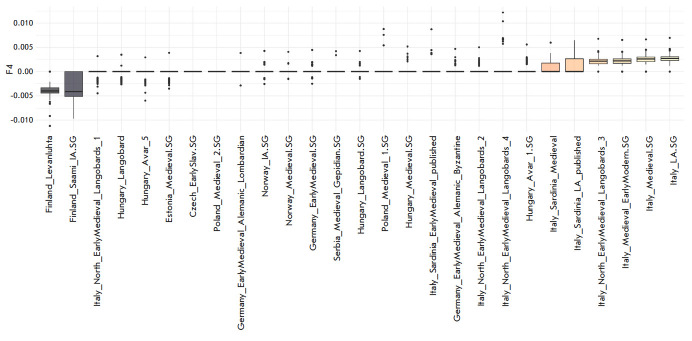
Values of F4-statistics in the form (“Vikings,” Test population; Bolshoy Oleny Island, Yoruba2). Test populations are plotted horizontally. The boxplot displays the F4-statistic values for the “Vikings” group. Statistically significant values are marked in gray for negative values of the F4-statistic and in yellow for positive values

We studied the contribution of the genome of people from Bolshoy Oleny Island to the formation of the medieval population living in the Baltic territories of modern Finland, Denmark, Sweden, and Norway. For this purpose, we used the genomes of the “Vikings” published in the 2020 Margaryan article [4] (we use this term not for historical purposes but for brevity of the reference to the population under study). All of these samples had a VK identifier and a digital code. F4-statistics of the form (VK, Test; Bolshoy Oleny Island, Yoruba) showed negative values (z > 10) only when the population of Finns (Finland_Levanluhta) or Saami were used as a test (Finland_Saami_IA.SG), but positive values were encountered when the population of southern Europeans was used as a test: for example, sample Italy_Medieval_EarlyModern.SG (z > 13). The results are presented in *Fig. 3* and in *Table 1* of Supplementary 9.

Thus, when comparing the VK–Saami pair, genes flow from Bolshoy Oleni Ostrov individuals to the Saami. But when comparing the VK–Southern Europe pair, a significant contribution of Bolshoyrelated ancestry in the populations of the “Viking” was detected. Most likely, this gene flow occurred through the contacts of the “Vikings” with the Finno- Ugric population of the Baltic region.

The unexpected similarity of the Y chromosomes of Prince Dmitry Alexandrovich and the ancient people from Bolshoy Oleny Island made it possible to hypothesize that the contribution of the East Eurasian genes might have been significantly higher in the “Vikings” with the N1a haplogroup compared to the “Vikings” with the R1a haplogroup. Indeed, F4- statistics in the form (Vikings_R1a, Vikings_N1a; Big Deer Island, Yoruba2) indicated a significant flow of East Eurasian genes into the “Vikings” with the N1a haplogroup (F4 = -0.00032, Z = -3.46). The results are presented in *Table 3*. At the same time, the genome of Prince Dmitry Alexandrovich did not show a significant difference in terms of the East Eurasian genetic component compared to other “Vikings” with haplogroup N1a.

**Table 3:** Values of F4-statistics in the form (Vikings_R1a, Vikings_N1a; Big Deer Island, Yoruba2)

H1	H2	X	O	D	Z
Viking_R1a	Viking_N1a	BolOlen	Yoruba2	-0.000315	-3.463
Viking_R1a	Viking_N1a	BolOlen	Yoruba	-0.000247	-2.737
Viking_R1a	Viking_N1a	BolOlen	Mbuti	-0.00019	-1.988

The hypothesis that the people from Bolshoy Oleny Island are one of the optimal proxy populations for four-ways admixture was tested by repeating the modeling that was performed by Margaryan et al [4]. Some Viking populations, such as Ladoga and Estonia_IA, could not be modeled as a mixture of three ancestral populations: European hunter-gatherers, Neolithic farmers, and steppe pastoralists (*Table 1* of Supplementary 10). To achieve a successful modeling, a fourth source was added, which was represented by the eastern samples of the Xiongnu Iron Age (about 100 BC–50 AD) or samples of the Bolshoy Oleny Island. It turned out that the Scandinavian populations were modeled equally effectively (*p *> 0.05) using both the Xiongnu [4] and Bolshoy Oleny Island samples (*Table 2* Supplementary 10). Their genetic contribution to these populations was as follows: to Ladoga – 4.7% Xiongnu and 4.7% Bolshoy Oleny Island; and to EstoniaIA – 6.5% Xiongnu and 8.4% Bolshoy Oleny Island.

Thus, it is clear that the gene pool of medieval “Vikings,” representing a significant part of Northern Europe (island and mainland), came into being partly through a flow of genes from Siberia, and the male ancestors of Prince Dmitry Alexandrovich were, with a high probability, men who left the Bolshoy Oleny burial ground island on the coast of the Kola Peninsula about 3,600 years ago.

**Analysis of mtDNA of Prince Dmitry Alexandrovich**

The mitochondrial haplogroup of Prince Dmitry Alexandrovich was identified as F1b1. This haplogroup refers to the East Eurasian cluster, and its representation at different frequencies in the gene pools of most of the previously studied ancient and modern populations of the Baikal region and adjacent territories of Central Asia is noted [35, 36, 37, 38]. Also, the mitochondrial haplogroup F was found in three Avars of the VII century in the Danube-Tiss interfluve (F1b1b and two samples with F1b1f). The genomic profiles of these individuals of the middle Avar period correspond to the genomes of other members of the elite of the early Avar period in this region and consist of 90–98% of the ancestral component AR_ Xianbei_P_2c, which has an eastern steppe origin and acts as a genetic component of the ancient northeast Asians (ANA). Two of the three burial places (male burial places) were characterized by a rather rich inventory of gold and gilded objects, which indicates their belonging to the nobility [39].

It is rather difficult to interpret the origin of the mitochondrial haplogroup of Prince Dmitry Alexandrovich, since for almost all historical epochs there is an increased variability and “diversity” of the mitochondrial composition of the female part of the groups of the ancient population. This is due to the fact that marriages of an official and unofficial nature concentrated representatives of completely different genetic lines in one geographical locus. When examining the history of dynasties, it is important to keep in mind that the attraction of women of various backgrounds as a beneficial or forced political move is a widespread phenomenon. Thus, the F1b mitochondrial group of Prince Dmitry Alexandrovich can be associated both with the ancient northern flow from the territory of Siberia (East Eurasian component) [34] and with migration of early medieval nomads [39], while the source of this group can probably be the same.

**Results of PCA analysis**

**Fig. 4:**
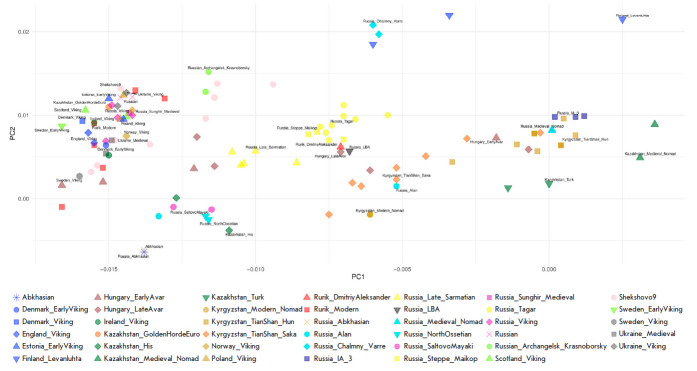
Principal component analysis. The genome of Prince Dmitry Alexandrovich was projected onto the ancient and modern populations of Eurasia

The principal component analysis (PCA) was used to assess the genetic affinities of the genome of Prince Dmitry Alexandrovich to other known ancient and modern populations. The results of the PCAanalysis for 740 samples are shown in the figure in Supplementary 11 (the list of samples is presented in *Table 1* of Supplementary 2). A simplified version of these results is reproduced in *Fig. 4* (only 116 samples are displayed, the list of samples is presented in *Table 2* of Supplementary 2). The location of ancient and modern genomes on the PCA map correlates with the geographical coordinates of the corresponding archaeological sites (Pearson correlation 0.76). The PC1 axis mainly coincides with the West– East direction; and the PC2, with the North–South. The genome of Prince Dmitry Alexandrovich (sample coordinates PC1: -0.0071, PC2: 0.0062) holds an intermediate position between the European and Central Asian clusters. The ancient samples closest in time to Prince Dmitry Alexandrovich belong to an early medieval population of Central Europe, the Avars steppe nomads of the late period; for example, the Hungary_ LateAvar (ID I16741) [40] and Hungary_Transtisza_ LAvar (ID ARK-11) [41].

The Avars were a nomadic people originating from Central Asia who moved to Central Europe in the 6th century and created the state of the Avar Khaganate there (VI–IX centuries). Archaeologists often define the Avars as Caucasoids, suggesting that only a small ruling stratum, the elite, retained a pronounced Mongoloid feature. The recently published genomes of ancient individuals of the Avar period demonstrate their genetic heterogeneity; on the principal component plot, the studied samples are scattered over the entire wedge from the populations of Western Eurasia to the populations of Northeast Asia [41]. Despite this heterogeneity, some patterns were identified: representatives of the early Avar elite form a dense cluster with a high content of the “Ancient Northeast Asians” (ANA) component, while the samples of the Late Avar period are shifted towards Western Eurasia. In turn, representatives of the Avars who are not associated with the elite are quite diverse and have a significantly smaller component of the “ancient northeast Asians” or it is completely absent. Hungary_Late Avar (I16741), an individual of the late Avar period with a mixed genomic profile consisting of ~20% of the Eastern Steppe component and ~80% of the component most pronounced in the previous local inhabitants of the Carpathian Basin clustered next to Prince Dmitry Alexandrovich, belongs to this group of samples [41].

**Admixture analysis**

**Fig. 5:**
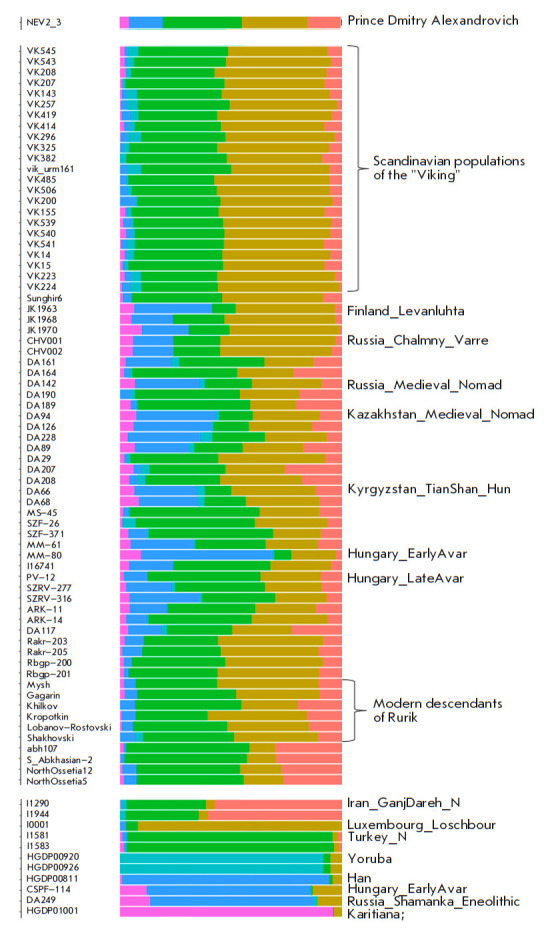
Results of the Admixture analysis for representatives of ancient populations whose genomes were used to assess the genetic origin of Prince Dmitry Alexandrovich (the number of ancestral populations is six (*K *= 6))

Analysis of the genetic origin of Prince Dmitry Alexandrovich was carried out using the Admixture method. Supplementary 12 presents the results of an Admixture analysis with parameters *K* from 6 to 12. The results of the Admixture analysis in simplified form with the number of ancestral populations equal to six (*K* = 6) are shown in *Fig. 5*. Representatives of ancient populations are shown whose genomes were used for modeling the genetic origin of the prince.

When decomposing the genome of Prince Dmitry Alexandrovich into ancestral components, it should be noted its genetic similarity to representatives of the early medieval population of the east of Scandinavia, the “Vikings,” which may militate in favor of the version of the “Vikings” (Scandinavian) origin of Rurik, the ancestor of the princely family called Rus’, which the Chronicle directly indicates. Here and below, we use the term “Vikings” in quotation marks to show that this is a heterogeneous and complex European population in its historical formation, united only by the way of life and habitat.

Comparison of the genome of Prince Dmitry Alexandrovich with the genomes of the Scandinavian populations of the “Viking” Age, including those from the territory of modern Russia [4], indicates the presence of an additional East Eurasian component in a significant amount (indicated in dark blue color). The indicated component is maximally expressed among the Nganasans, an indigenous people in Siberia, the Finno-Ugric Mansi people, representatives of the indigenous Han people in China (East Asia), among the Avars elite from the Danube-Tisza Interfluve (Hungary_DanubeTisza_ MLAvar) [41], as well as among the Early Neolithic of Lake Baikal (Russia_Shamanka_Eneolithic.SG) [42] and Mongolia (Mongolia_North_N). To a lesser extent, this component is present in samples of the early Middle Ages from the territory of modern Finland (Finland_Levanluhta), in a sample synchronous with Prince Dmitry Alexandrovich from the Caspian steppe (Russia_Medieval_Nomad), as well as in more ancient Iranian-speaking steppe nomads of the Iron Age from the territories of modern Kazakhstan and Kyrgyzstan (Kazakhstan_TianShan_ Saka, Kyrgyzstan_TianShan_Hun). Due to the fact that the steppe and Finno-Ugric populations share a common origin, this type of analysis does not allow us to specifically attribute this component to one of these groups, with all used *K* equal to 6–12.

Thus, based on PCA data, Admixture analysis, and information on mitochondrial DNA, it can be argued that Prince Dmitry Alexandrovich had a significant eastern component in his genome. This distinguishes him from the early medieval population of the east of Scandinavia, the “Vikings,” and the medieval Slavic sample from Vladimir (Sungir6), but it makes him closer to the ancient population of Finland, the Kola Peninsula, and the early medieval population of central Europe, which includes a well-known component of steppe nomads. Probably, this contribution came from both the male and female lines, which corresponds to the routes of ancient migration from Siberia to the north of Europe and migrations from Siberia in the first millennium BC – the first millennium AD along the Eurasian steppe corridor.

**Modeling the genome of Prince Dmitry Alexandrovich from the genomes of ancestral populations**

After analyzing the results of the PCA and Admixture analysis, as well as available historical information, we selected populations that could participate in the formation of the genome of Prince Dmitry Alexandrovich: the genomes of the early medieval population of the east of Scandinavia; representatives of the Iranian-speaking nomads of the Eurasian steppes of the Iron Age, and the population of the early Middle Ages of Central Europe, which includes a well-known component of the steppe nomads and samples of individuals representing the ancient East Eurasian component. To assess the contribution of the Slavic component to the genome of Prince Dmitry Alexandrovich, samples of the Medieval Russian population of the XI century from the rural necropolis of the Shekshovo settlement in Suzdal Opolye and an individual of the XII century from the territory of modern Vladimir (Sungir6) were used [43, 44]. Several models were tested:

**Fig. 6:**
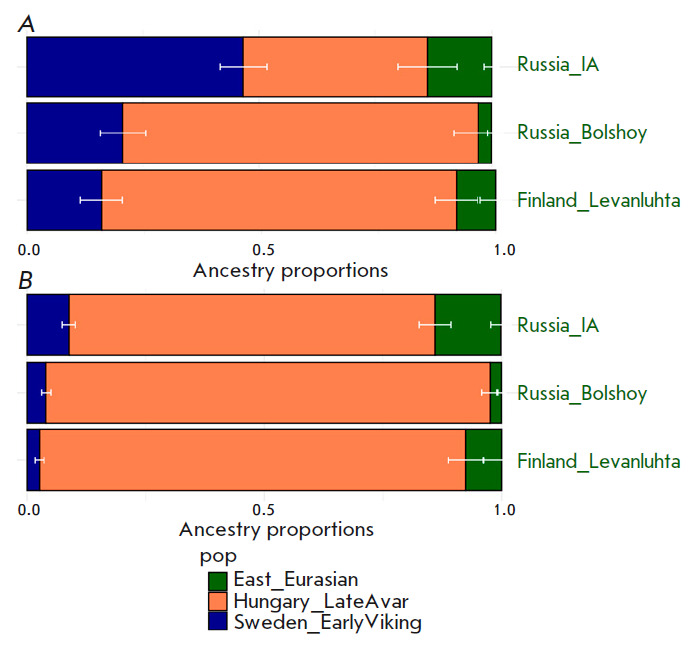
The results of modeling the genome of Prince Dmitry Alexandrovich as a three-way admixture of “Vikings” (dark blue color), the early medieval population of Central Europe (orange), which includes a well-known component of the steppe nomads, and the ancient East Eurasian component (green). Early “Vikings” from the territory of Sweden (*A*) and early “Vikings” from the territory of Estonia (*B*) are shown as representatives of the “Vikings”

*Modeling of the genome of Prince Dmitry Alexandrovich using the genomes of the “Vikings”.* QpWave-analysis determined that a minimum of three ancestry sources were sufficient to model the genome of Prince Dmitry Alexandrovich (Supplementary 13). Exploring all possible combinations of three sourc es from the selected list was accomplished using the qpAdm tool. Results of the modeling of the genome of Prince Dmitry Alexandrovich (*p* greater than 0.05) are presented in Supplementary 14 (*Table 1*). The genome of Prince Dmitry Alexandrovich can be successfully modeled as a three-way admixture of the “Vikings,” steppe nomads, and ancient East Eurasian components. For example, one of such models includes 46.6% of the early medieval population of the east of Scandinavia (Sweden_EarlyViking); 39.6% of the early medieval population of central Europe, which includes the known component of the steppe nomads (Hungary_LateAvar); and 13.8% of the Russia_IA source as the third component (Iron Age sample from the territory of the Republic Altai). Substitution of Russia_IA with Iron Age samples from the territory of Finland (Finland_Levanluhta) or the Bolshoy Oleny Island in the Kola district of the Murmansk region produced a reliable three-way admixture model. These third components (Russia_IA, Finland Levanluhta and Bolshoy Oleny) have the ancient East Eurasian component in common. According to recent studies, people of that ancestry arrived in the Kola Peninsula more than 3,500 years ago from Siberia [34] and mixed with local populations thus forming people who speak the Finno-Ugric language today. The results are presented in *Fig. 6A* and in Supplementary 14 (*Table 1*).

**Fig. 7:**
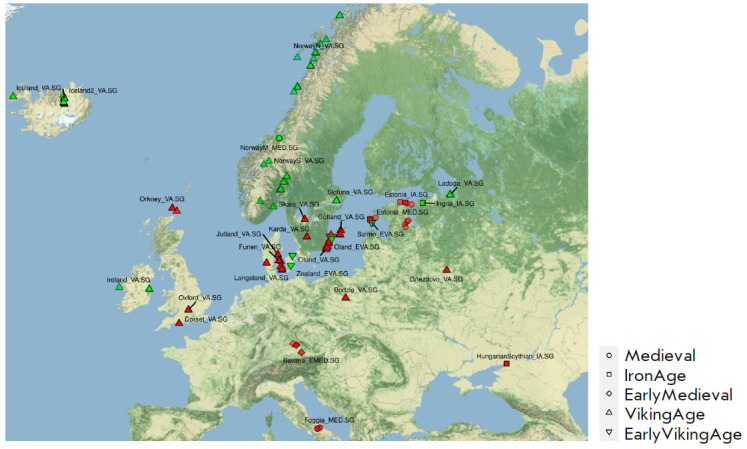
The map shows the burial sites of “Vikings” who confirmed (green) or did not confirm (red) the model of one source for the Sungir6 sample. The shape of the label reflects the temporal classification of images; the decoding is given in the legend to the right of the figure.

Among “Vikings” Sweden_EarlyViking was the largest contributing population to the genome of Prince Dmitry Aleksandrovich (46.6%). For other “Vikings” this proportion did not exceed 9%. The minimum contribution is made by the Estonia_ EarlyViking population: 2.7% (*Fig. 6B*). The Sweden_ EarlyViking population is represented by three samples from the village of Bode (Böde) on the island of Öland and dated around the period between the VII and VIII centuries AD (with ID’s VK379, VK382, VK359). The analysis of strontium isotopes for these samples [4] attributed them to the category of migrants to the town of Bode, although the question of whether they were the original inhabitants of the island of Oland remains open.

Our qpAdm analysis showed a significant genetic difference between the Oland_Sweden_EarlyViking group and other samples from Oland Island, designated as Oland_Sweden_Viking: the first one can be successfully (*p*= 0.64) modeled as a three-way admixture between European hunter-gatherers, Neolithic farmers, and steppe pastoralists (Supplementary 14 *Table 2*), while the Oland_Sweden_Viking group could not be modeled as a mixture of these ancestral populations (*p* = 0.01). There are also significant differences in the modeling of these two groups of “Vikings” using a single source group, which are Iron Age populations from the territory of Europe or a sample of the Medieval Russian population of the XI century from the territory of modern Vladimir, Russia (Supplementary 14 *Table 3*). In an extended analysis, all “Viking” population groups were used to test the single-source model for the Sungir6 sample (*Fig. 7*, Supplementary 14, *Table 4*). Those populations of “Vikings” that provide reliable values of F4-statistics for this model are concentrated in the northern part of Europe, Ireland and Iceland, while none of the southern populations fits into such a model. These results raised the question of the relationship of the Scandinavian population groups to the Slavs in the period from the VI to the XI centuries.

**Modeling the genome of Prince Dmitry Alexandrovich using the genomes of Iranian-speaking nomads of the Eurasian steppes in the Iron Age**

**Fig. 8:**
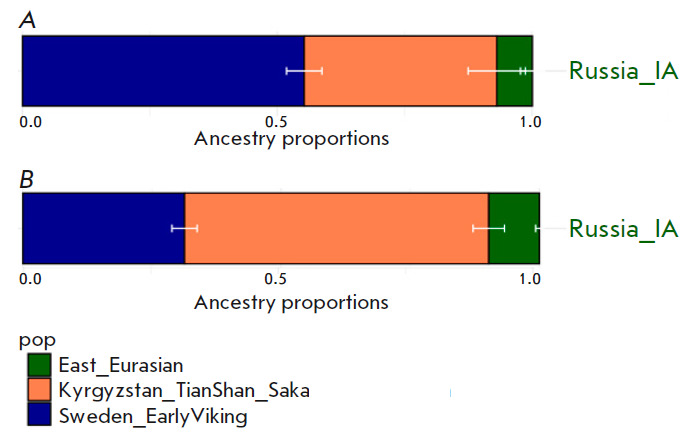
The results of modeling the genome of Prince Dmitry Alexandrovich as a three-way admixture of “Vikings” (dark blue color), ancient East Eurasian component (green), Early Iron Age nomads from the territory of Kyrgyzstan (*A*) and Kazakhstan (*B*) (orange)

A statistically reliable genetic model of the genome of Prince Dmitry Alexandrovich was also obtained by replacing the early medieval population of Central Europe (including a component of steppe nomads) with Iranian-speaking nomads of The Eurasian steppes in The Iron Age (Kazakhstan_TianShan_Saka, Kyrgyzstan_TianShan_Hun). The results are shown in *Fig. 8* and in Supplementary 14 (*Table 5*). The model provides a fits only with early Iron Age nomads from the Tien Shan region, perhaps, due to their genetic profile: high proportion (70%) of the Late Bronze Age steppe pastoralists, 25% of the South Siberian hunter-gatherer component, and 5% of the component associated with the Neolithic population of Iran [45]. This group of nomads, according to the results of f3-outgroup-statistics, is genetically closer to northern European populations compared to other nomads of the early Iron Age from the Asian cluster.

**Modeling the genome of Prince Dmitry Alexandrovich using the genomes of the Medieval Russian Slavic population**

We hypothesize that alternative models – replacing the population of “Vikings” with Medieval Russian Slavic populations – will likely also provide a fit. Since the pagan Slavic tradition practiced cremation until the end of the X century, we used samples of the medieval Russian population of the XI century from the rural necropolis of the settlement of Shekshovo9 in the Suzdal Opolye and an individual of the XI century from the territory of modern Vladimir, Russia (Sungir6) [42, 43]. The genomes of individuals from Shekshovo9 are the result of a mix of the Central European (Slavic) and local (Finnish) genetic components, while Sungir6 is considered a pure Medieval Russian Slavic population. These features of the samples are confirmed in the PCA plot: Sungir is located in a cluster of European (Danish, Polish, Norwegian, Ukrainian, etc.) medieval samples, and Shekshovo9 is shifted to the “East” along the PC1 axis (*Fig. 4*).

**Fig. 9:**
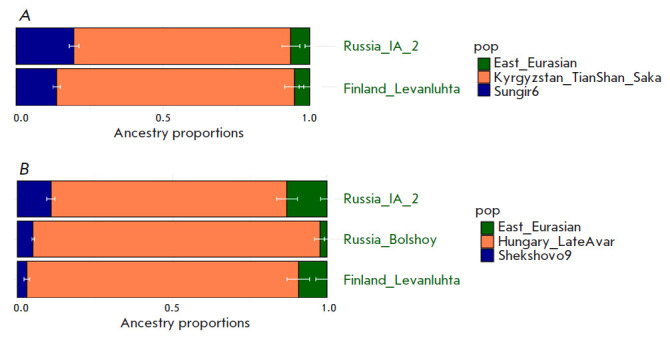
The results of modeling the genome of Prince Dmitry Alexandrovich as a three-way admixture of the Medieval Russian Slavic population (dark blue color), the ancient East Eurasian component (green), and (*A*) representatives of the steppe nomads (orange) of the Early Iron Age or (*B*) the early medieval population of Central Europe (steppe nomads from the territory of Hungary)

We successfully modeled Prince Dmitry Alexandrovich’s ancestry as being derived from populations related to the Sungir6 sample – 19.7%; Iron Age nomadic steppe peoples – 73.8% for Kyrgyzstan_ TianShan_Saka; and the Iron Age from the territory of the present-day Altai Republic – 6.5% for Russia_IA (*Fig. 8* and in Supplementary 14, *Table 6*). Replacing the Sungir6 with Shekshovo9 as a source, the proportion of the Medieval Russian Slavic population amounts to 18.7%; the nomadic Iron Age steppe peoples (Kyrgyzstan_TianShan_Saka) – 78.2%; and Russia_IA_2 – about 3% (*Fig. 9A*). In the slightly different three-source modeling with the Hungarian Avars, Slavic Medieval Russian and Russia IA – the proportion reached 76,2%, 10,8%, and 13,1%, correspondingly (*Fig. 9B*). The decrease in the Slavic proportion can be explained by the partial compensation of Slavic origin by the Avars individuals who were used for modeling. Their origin suggests 80% of the local East European source and only 20% of the Central Asian one (ID I16741) [40].

Thus, modeling of the genome of Prince Dmitry Alexandrovich indicates the contribution of three ancestral sources to its origin: (1) the early medieval population of the east of Scandinavia from the island of Oland, (2) the steppe nomadic peoples of the Eurasian steppes of the Iron Age or the early medieval population of central Europe (steppe nomads from the territory of Hungary), and (3) the East Eurasian component. Alternative models, replacing the population of “Vikings” with the Medieval Russian Slavic populations (Shekshovo9 and Sungir6) also provide a fit.

## CONCLUSION

Paleogenetics and mathematical statistics provided an opportunity to discuss the origin of the Rurikids and design a reliable tool to attribute remains with disrupted documentation to this ruling family.

An analysis of the genealogical tree of the Rurikids showed that the modern individuals of this family, who have a Y chromosome clustered with Prince Dmitry Alexandrovich’s Y chromosome, belong to three different branches – the Olgoviches, Mstislaviches, and Yuryeviches. Thus, the N1a haplogroup of the Y chromosome characterizes all three branches of the tree, suggesting that their first common ancestor, Prince Yaroslav the Wise, was a carrier of N1a haplogroup also.

The mitochondrial haplogroup of Prince Dmitry Alexandrovich was determined as F1b1, which may point to the contribution of eastern populations to his genome. This hypothesis is also supported by autosomal data (PCA and Admixture). Although the main genetic makeup of Dmitry Aleksandrovich can be attributed to the Scandinavians/Slavic/European populations our results provide clear evidence of the input of the Eastern genetic component. This is in line with historical data: marriages of Russian princes with the daughters of the Polovtsian khans from the end of the XI century were a common practice that cemented allied relations and political interaction [46, 47]. Dmitry’s mother, the wife of Alexander Nevsky, Alexandra Bryachislavna, came from the Polotsk Izyaslaviches family. Information about the wives of these princes is scarce, and the name and origin of Alexandra’s mother is unknown. However, men of the Polotsk branch of the Rurikids did not avoid marriage alliances with Polovtsy women. From the Polovtsian family came the second wife of the Polotsk Prince Svyatopolk Izyaslavich (1050–1113), Elena, the daughter of Khan Tugorkan (Complete collection of Russian chronicles, 1997, vol. I, p. 231–232). The circulation of eastern mitochondrial groups in this situation is quite expected. Alternatively, the origins of the eastern component in the genome of Dmitry Alexandrovich might be associated with the marriages of the Rurikids with representatives of the dynasties of Central and Southern Europe (Serbian Vukanovichi, Hungarian Arpads). Eastern genes brought by the migration of the first millennium AD [48] could be much better preserved within elites than within plebs.

Our results raise some questions on Rurikids’ genetic history. The most obvious of them are (i) how to explain the presence of the “Eastern component” in the genome of Dmitry Aleksandrovich and (ii) were there a genetic connection between Scandinavians and Slavs in the pre Rurik era? The answers may come after a systematic paleogenomic study of new, reliably documented paleoanthropological materials from the territory of Russia.

## Abbreviations

aDNA: ancient DNA
SNP: single-nucleotide polymorphism
PCA: principal component analysis
